# Supplementary material for: Microbial paracetamol degradation involves a high diversity of novel amidase enzyme candidates
Source: Water Res X. 2022 Aug 4;16:100152. doi: 10.1016/j.wroa.2022.100152 (PMC9420511; doi:10.1016/j.wroa.2022.100152)
Supplement: Supplementary file 2 [file mmc2.docx]

CLUSTAL O(1.2.4) multiple sequence alignment

Based on the crystal structure of ACP39716.2 in a soil bacterium, we marked the following regions:

- Canonical catalytic triad: Ser^187^-*cis*Ser^163^-Lys^84^.
- Gly/Ser-rich motif: **GGSSGG**.
- The oxyanion hole: **[G]GGS**.
- Loop 1: Asp^85^ – Ser^114^.
- Loop 2: Pro^202^ – Leu^222^.
- *α-helix* (α10): Asp^354^-Val^363^
- Substrate-binding residues: Tyr^136^ and Thr^330^.

Unbinned_Comamonadaceae_KGEGGEOM_08132 ----MTNVNSDVIDLTELTVERVQAGFASGAFTSETLTKAYLKRIEEFNPSYNAIVFFN- 55

Paracoccus_huijuniae_AFC37599.1_AmpA ---------MIPRLTNEPGAIEVAAQIRAGELSPLEAANAAIARIEALDGPLNAVVVRDF 51

Ochrobactrum_sp.TCC-2_ANB41810.1_TccA --------MTATDELYYLPVTELSELIAERRLAPSELMSAVIARAEEVNPKLNALVAQRF 52

SLOW-growing_Pseudomonas_BKDBLJDL_05334 --------MTQKTAIGELTAVELLELYQRRQLSPVEVVDDVLARIDLHNPAVNAYCHVDG 52

Unbinned_Actinomycetia_KGEGGEOM_07080 ---------MDPSEYAWMSATDLVKALQSKQLSPVDLIDAAIARIEQRNPAINAVVHLGA 51

Unbinned_Actinomycetia_KGEGGEOM_08489_partial ------VIDHRNLPNRWVSATETVRRITSGEQSATEVTTDSIHRIEHLNSMYNAVTFSDP 54

Microbacterium_BJLACCKG_02946 VNADPVSIASRHTAGAANTATETARRVRAGEISALEVTEEALARIDQVNPALGAVTFVAA 60

Ochrobactrum_sp.PP-2_ANS81375.1_Mah ---------MERSDLDYASATEIARLVRTRQISAADVTEHAISRIEARNGSLNAFVYTDF 51

FAST-growing_Pseudomonas_OACKLNDA_05760 ---------MIGSQVHWKTATEIAGLVKSKQISPREVVEGTIELIEQRNPSMNAVTYKAY 51

Crystal_structure_bacterium_CSBL00001_ACP39716.2 -------MGKSHSPVHWKSAAEIVELVKSKQISPREVVESTIDLIEQRDPGLNAVVYKAY 53

. : : : : .*

Unbinned_Comamonadaceae_KGEGGEOM_08132 EKAVEEARAIDARRAAGEKLGPLAGVPVVVKEAMD-MKGFPTTGGWSLLYSKTGGVDLLP 114

Paracoccus_huijuniae_AFC37599.1_AmpA DRARDAARELDGQPA---EDRPLFGVPMTVKESFD-VAGLPTTWGHVPFKDY---RPT-- 102

Ochrobactrum_sp.TCC-2_ANB41810.1_TccA EAATREAAAADN----EPSRGVLHGIPITLKDLAYETPDLPSTYGSRAFAGY---EP--- 102

SLOW-growing_Pseudomonas_BKDBLJDL_05334 EGARAAARASEQRWQRGQPCGRLDGVPASIKDLTL-TRGMPTRKGSRTTS-----GSGPW 106

Unbinned_Actinomycetia_KGEGGEOM_07080 EDARAAARTAERALA-EGRAGPLAGLPVLMKDLFDFKPGWPSTLGGVPALAD---NIAQ- 106

Unbinned_Actinomycetia_KGEGGEOM_08489_partial DLALSTARDIDRRLAASDEVGPLAGVPTLMKDLYGFRPGWPTTLGGLSAARG---DTASE 111

Microbacterium_BJLACCKG_02946 DQARDAAARLDRRIRSGESVGPLAGVPTLVKDLYGFVPGWPSTLGGIEALRD---HRTPD 117

Ochrobactrum_sp.PP-2_ANS81375.1_Mah EQARSRAKDLDTRISAGEDVGPLAGVPTAIKDLFNFYPGWPSTLGGIRCLRD---FKL-- 106

FAST-growing_Pseudomonas_OACKLNDA_05760 DEARVKAAELERHIMNGGRTGALAGVPTLMKDLFAAKPGWPSTLGGISALKH---LRGAE 108

Crystal_structure_bacterium_CSBL00001_ACP39716.2 DEAREKAAALERRIMQGEPVGMLAGVPTLMKDLFAAKPGWPSTLGGIRALKD---ARGAA 110

: * * : * *:* :*: . *: *

Unbinned_Comamonadaceae_KGEGGEOM_08132 ETDSPVVARMRQADTVILGKTNIPILSHTGAHANGSWAGSTYNSAGREFLP**GGSS**A**G**TAT 174

Paracoccus_huijuniae_AFC37599.1_AmpA -RDARVVQLLKDAGAIILGKTNVPPDLADMQSNNP-VYGRTNNPYDHSRVA**GGSSGG**SAV 160

Ochrobactrum_sp.TCC-2_ANB41810.1_TccA GFETVVGQRLRAAGTIAIGRTNSPEFGLTNTCESA-QFGPTSNPWRPEHTP**GGSSGG**AGA 161

SLOW-growing_Pseudomonas_BKDBLJDL_05334 EIDAPFSAFMREAGAVLVGKTTTPEFGWKGVTDNP-LYGITRNPWDTRLTA**GGSSGG**AAA 165

Unbinned_Actinomycetia_KGEGGEOM_07080 -HSCLFVERMESAGAIVLGKTNSPVFGARGITDNP-LFGPTCNPFDVSRNP**GGSSGG**AAA 164

Unbinned_Actinomycetia_KGEGGEOM_08489_partial GAWSRFPKQVTADDGVLLGQTNSSTFGFSGVTDNA-LFGPSRNPFEPTRNT**GGSSGG**SAA 170

Microbacterium_BJLACCKG_02946 GLWSAYPRSAVSADAILIGQSNSPVFGFRGVTDNT-LFGPTRNPFDLTRNA**GGSSGG**AAA 176

Ochrobactrum_sp.PP-2_ANS81375.1_Mah DVKSRYATKMEEAGAVVLGITNSPVLGFRGTTDND-LYGPTRNPFDLSRNS**GGSSGG**TSA 165

FAST-growing_Pseudomonas_OACKLNDA_05760 GAWSTFPLKMSNEDSLLLGQTNSPVFGFRGVTDNK-FFGPTRNPFNLDYNA**GGSSGG**AAA 167

Crystal_structure_bacterium_CSBL00001_ACP39716.2 GVWSTYPLKMSGEDSLLLGQTNSPVYGFRGTTDNT-FFGPTRNPFNLDFNA**GGSSGG**AAA 169

. : :* :. . * : * ****.*:..

Unbinned_Comamonadaceae_KGEGGEOM_08132 AVGANFCVLGLAEETA**GS**IQNPASAQGLVGIKPTFGLVPNAGVMP--------LASLRDV 226

Paracoccus_huijuniae_AFC37599.1_AmpA AVATGMVPAEYGSDI**G**S**S**IRNPAHFNGIYGHKTTFGLVSRRGHGHPVAGGKDMHAGPLSV 220

Ochrobactrum_sp.TCC-2_ANB41810.1_TccA AVAAGIAPLAAAND**GGGS**CRVPASSCGVVGLKPSRGRVPWAPTSY-----EYW--AGFAT 214

SLOW-growing_Pseudomonas_BKDBLJDL_05334 AAALNLGVLHQGSDA**GGS**IRIPCAFTGTFGIKPTFGYVPQWPASA-----MTV----LSH 216

Unbinned_Actinomycetia_KGEGGEOM_07080 AVAAGFVPIAEGTDA**GGS**IRIPAAWTSTYGFKPSAGRVPSIIRPL-----GFMTAAPFIT 219

Unbinned_Actinomycetia_KGEGGEOM_08489_partial AVASGMIPVAGASDA**GGS**IRIPAAWTNTVGFQPSAGRVPSTPRPA-----LFHL-GPHLY 224

Microbacterium_BJLACCKG_02946 AVATGIVPVAGASDA**GGS**IRIPAAWTNTVGFQPSAGRVPSTPRPV-----GFHL-APFLY 230

Ochrobactrum_sp.PP-2_ANS81375.1_Mah AVADGLLPIGDGTD**GGGS**IRIPAAWCHVFGFQASPGRIPLAIRPN-----AFGAAAPFIY 220

FAST-growing_Pseudomonas_OACKLNDA_05760 VVADGIVPIAGGTDA**GGS**VRIPAAWTNTYGFQPSIGRIPFISRPN-----AFHL-ATYIY 221

Crystal_structure_bacterium_CSBL00001_ACP39716.2 LVADGIVPVAGGTD**GGGS**IRIPAAWTNTYGFQPSIGRVPFKSRPN-----AFHP-GPYLY 223

.. .: . : ..* : *. * : : * :

Unbinned_Comamonadaceae_KGEGGEOM_08132 VGPIARCVRDAALTLDVLAGFSMEDPKTNASVGRRPKGGYASKLDKGALAGKRIGLYGP- 285

Paracoccus_huijuniae_AFC37599.1_AmpA TGPLARSAEDLQLLLQVTAERP-------------------LARRRKSLTEMRFLAVLD- 260

Ochrobactrum_sp.TCC-2_ANB41810.1_TccA NGPIARTVEDVALLLDAMSGPVVGEPYGLP-----APSESFLTASRRRPGPLRIAFSCTP 269

SLOW-growing_Pseudomonas_BKDBLJDL_05334 LGPMTRTVDDSVLMLDCVARPDARDGLAGA-----PRQAPWLSQQ-QDLSGLRIAYSAN- 269

Unbinned_Actinomycetia_KGEGGEOM_07080 EGPITRTVADAALAMTALAGPDSRAPHCLD-----GVLDYRAALD-GSIAGRRLGYSPN- 272

Unbinned_Actinomycetia_KGEGGEOM_08489_partial EGPITRTVEDAVLLMNSLQGYDPRDPYAAP-----APAFSPALLS-SGVRGKRIGLSLD- 277

Microbacterium_BJLACCKG_02946 EGPITRTVQDAALVIDALSSHDPHDPTSVD-----GLPSMSEAIT-RDIDELRIGIVED- 283

Ochrobactrum_sp.PP-2_ANS81375.1_Mah EGPITRTVEDAALAMSVLAGSDPADPFSLN-----DRLDWLGAVD-QPITSLRIGFTPD- 273

FAST-growing_Pseudomonas_OACKLNDA_05760 EGPITRTVEDAALAMNALHGFDRRDPNSLR-----VKLDFTSALV-QGVRGKKIGLTLD- 274

Crystal_structure_bacterium_CSBL00001_ACP39716.2 EGPITRTVRDAALAMNVLHGFDRRDPASLR-----VKLDFTSALA-QGVRGKKIGLTLN- 276

**::* . * * : ::

Unbinned_Comamonadaceae_KGEGGEOM_08132 GWRNSTFGEETISLYARAQEELKKLGATFVNDPFAGSGLRELRKPIMPGAEFDARGMESI 345

Paracoccus_huijuniae_AFC37599.1_AmpA -HPSSAIDASVRGPIEAALAEIERAGASVDRAS------------------------ALL 295

Ochrobactrum_sp.TCC-2_ANB41810.1_TccA PKPHDRLNAEVKQTFLAAVANFEALGHTVTEID------------------------HGL 305

SLOW-growing_Pseudomonas_BKDBLJDL_05334 -FGYVQVAPQIQALVAQAVQRLARLGAQVEEVD------------------------PGF 304

Unbinned_Actinomycetia_KGEGGEOM_07080 -LGAFPVEPAVARAVEHALTGFEECGAQVQQTE------------------------VSL 307

Unbinned_Actinomycetia-KGEGGEOM_08489_partial -FGGFPVNPLVRDTITEAAATFEQLGAIVDPID------------------------VSL 312

Microbacterium_BJLACCKG_02946 -FGGFPVDPAVRATVRRAADAFSGVAKSVSAVS------------------------LNL 318

Ochrobactrum_sp.PP-2_ANS81375.1_Mah -FGGFPVEPAVAATIAHAVRAFEQAGAKIVPLK------------------------LDF 308

FAST-growing_Pseudomonas_OACKLNDA_05760 -YGVFPVQPEIKDLISKTAQVFTQLGAHVEFVD------------------------LGI 309

Crystal_structure_bacterium_CSBL00001_ACP39716.2 -YGVFPVQQEIQDLIGKAARVFTELGAHVEFVD------------------------LGI 311

. : : . . :

Unbinned_Comamonadaceae_KGEGGEOM_08132 PYDIEKYLQRMGANVALKTFADFAKATEKEGAFGPNGVLSYMPHSPEFV-EVMKN-P--- 400

Paracoccus_huijuniae_AFC37599.1_AmpA PDLAEQHYN-YMRLVNVAMMRGNPG------PLAEQHANYMR----LVNVAMMRGNPGPA 344

Ochrobactrum_sp.TCC-2_ANB41810.1_TccA DGIFDSFIR----VIAANTALSVTQ------TVPLGSLNLLE--PNTLGLAQRGW-G--- 349

SLOW-growing_Pseudomonas_BKDBLJDL_05334 SDPLETFNTLWFAGAARLASAL-------------------SDEQKALLDPGLRW-IAEQ 344

Unbinned_Actinomycetia_KGEGGEOM_07080 PADHEELAALWHRSMMQVTLASL-E------GMQAQGIDLLADLAAHFPPALAEQ-IEQ- 358

Unbinned_Actinomycetia_KGEGGEOM_08489_partial TYTHDELTQMWLRSMGLLMLADL-D------SLRARGRAL-A--PRDLPEPVLYW-TEV- 360

Microbacterium_BJLACCKG_02946 GQSHDELTETWLRMMGTAMLSEM-D------SHRRQKVDLTA--AGGVPLEVLRW-TSR- 367

Ochrobactrum_sp.PP-2_ANS81375.1_Mah GYTHDELSQLWCRMISQGTIAVV-D------SFAENGLHL----EPDFPAPVMEW-AQK- 355

FAST-growing_Pseudomonas_OACKLNDA_05760 PYSQEQMSDAWCRMLAIPTAASM-H------ALHEDGIDLFSEHRADIPDALMKW-IDA- 360

Crystal_structure_bacterium_CSBL00001_ACP39716.2 PYSQKQMSDAWCRMIAIPTVASM-Q------ALRKEGIDLYGEHRADIPDALMKW-IDA- 362

. .

Unbinned_Comamonadaceae_KGEGGEOM_08132 ---SNPPD-MASFIALRETYLDIFNSVFDKHKLDAV----IYPQMRGPLGPLHGDEV--- 449

Paracoccus_huijuniae_AFC37599.1_AmpA GQTMALADYYQLLDTQE-CNRYAWADLFAEYDFVLAPPLPFVAYPHDATPIYERRIPING 403

Ochrobactrum_sp.TCC-2_ANB41810.1_TccA ---LSAMDYCEAINHLRTTAALSMARWTEDFDVLLTPTLTDLPPLTGQMPSYDGDL---D 403

SLOW-growing_Pseudomonas_BKDBLJDL_05334 GAQISLGE-YTQALEARAELIAKMNAFHQRYDVLVSPMLPL---VAFEAGHNVP----PG 396

Unbinned_Actinomycetia_KGEGGEOM_07080 TRTMTARQ-VRIDDVLRTEIYETLTSAINSFDLLLTPTVGGLPVVNASRGETIGPTQLNG 417

Unbinned_Actinomycetia_KGEGGEOM_08489_partial AAAMSAAD-VRSDRVMRTAVLDGLVDAMENHDLLVGPTVVDLPVLNSSDELTIGPTTVDG 419

Microbacterium_BJLACCKG_02946 AMAMTMRE-LLADRETRTAVLDGFLSAMDGVDLLVGPTVTALPVLNSTGGRTVGPSEVDG 426

Ochrobactrum_sp.PP-2_ANS81375.1_Mah AKNATPLD-LHRDQVMRTKVYDVLNAAFSQVDLIAGPTTTCLPTPNGERGMTVGPSEIAG 414

FAST-growing_Pseudomonas_OACKLNDA_05760 VADINVQQ-ISADQILRTSVFDCMNRVFDRFDLLLAPTLACMPVRNATDGSTEGPSAING 419

Crystal_structure_bacterium_CSBL00001_ACP39716.2 VADISVQQ-ISADQLLRTTVFDCMNGVFDRFDLLLAPTLACMPVRNATDGCTEGPSQING 421

: . ..

Unbinned_Comamonadaceae_KGEGGEOM_08132 ----IDA-----MVVSEINIAGLPAVTVPAGFYASGAPFNLVIVAPQWSEADILALAYAY 500

Paracoccus_huijuniae_AFC37599.1_AmpA KDSAFADALAWAG---LANFPNLPSTVVPVGES-AGLPCGMQVMGPEWSDLDCIAAAGAI 459

Ochrobactrum_sp.TCC-2_ANB41810.1_TccA --ACYLHMLGHNAFTYPFNVTGQPALSIPCGWSTSGLPIGLQIIGGMGQEARVLALAAAY 461

SLOW-growing_Pseudomonas_BKDBLJDL_05334 --SGMAQWMEWTPFSYPFNLTQQPAASVPCGFTREGLPVGLQVVAGRFADEQVLRVCKVY 454

Unbinned_Actinomycetia_KGEGGEOM_07080 --QAVDPMIGWAL-TFPFNFTGHPAASVPAGLV-DGLPVGMQLIGRQMGDLDVLTFSAAY 473

Unbinned_Actinomycetia_KGEGGEOM_08489_partial --TAVNPLIGWCP-TYLTNFSGSPSVSVPAGFA-EGLPVGMLIIGRKHRDEDVLAAAAAL 475

Microbacterium_BJLACCKG_02946 --VPVDPLIGWCP-TYLTNFTGMPSISLPAGFA-ENLPVGLLIIGRKYRDAEVFTAAAAF 482

Ochrobactrum_sp.PP-2_ANS81375.1_Mah --TPINRLIGFCP-TFLTNFTGNPAASLPAGLA-DGLPVGLMLIGPRRDDLTVLSASAAF 470

FAST-growing_Pseudomonas_OACKLNDA_05760 --EKVNPLIGWCM-TYLTNFSGHPSASVPAGLI-DGLPVGMLIIGDRQADLDVIAASAAF 475

Crystal_structure_bacterium_CSBL00001_ACP39716.2 --EEIDPLIGWCM-TYLTNFSGHPSASVPAGLI-DGLPAGMLIIGDRQADLDVIAASAAF 477

*. *: :* * . * .: ::. : : . .

Unbinned_Comamonadaceae_KGEGGEOM_08132 EQGTTHRKAPELKKA-------- 515

Paracoccus_huijuniae_AFC37599.1_AmpA GALMEG----------------- 465

Ochrobactrum_sp.TCC-2_ANB41810.1_TccA EEAHPWAARKPPL---------- 474

SLOW-growing_Pseudomonas_BKDBLJDL_05334 EQHYPSRHLQAPITG-------- 469

Unbinned_Actinomycetia_KGEGGEOM_07080 ERARPWVGNYAQVDG-------- 488

Unbinned_Actinomycetia_KGEGGEOM_08489_partial ETAR------------------- 479

Microbacterium_BJLACCKG_02946 EKIRPWHGAYREIAIGNASVQPD 505

Ochrobactrum_sp.PP-2_ANS81375.1_Mah ERVQPWADSYRIPAARPLGSQ-- 491

FAST-growing_Pseudomonas_OACKLNDA_05760 EEARPWLQYYDIPARRALQ---- 494

Crystal_structure_bacterium_CSBL00001_ACP39716.2 ERASPWSQYYDIPAGRPL----- 495
